# Enhanced Extracellular Production and Characterization of Sucrose Isomerase in *Bacillus subtilis* with Optimized Signal Peptides

**DOI:** 10.3390/foods11162468

**Published:** 2022-08-16

**Authors:** Dan Guo, Mingyu Li, Mengtong Jiang, Guilong Cong, Yuxin Liu, Conggang Wang, Xianzhen Li

**Affiliations:** School of Biological Engineering, Dalian Polytechnic University, Dalian 116034, China

**Keywords:** isomaltulose, sucrose isomerase, cloning and expression, biochemical characterization, signal peptides

## Abstract

Sucrose isomerase (SIase) catalyzes the hydrolysis and isomerization of sucrose into isomaltulose, which is an important functional sugar widely used in the food industry. However, the lack of safe and efficient expression systems for recombinant SIase has impeded its production and application. In this study, enhanced expression of a SIase from *Klebsiella* sp. LX3 (referred to as KsLX3-SIase) was achieved in *Bacillus subtilis* WB800N, by optimizing the signal peptides. First, 13 candidate signal peptides were selected using a semi-rational approach, and their effects on KsLX3-SIase secretion were compared. The signal peptide WapA was most efficient in directing the secretion of KsLX3-SIase into the culture medium, producing a specific activity of 23.0 U/mL, as demonstrated by shake flask culture. Using a fed-batch strategy, the activity of KsLX3-SIase in the culture medium was increased to 125.0 U/mL in a 5-L fermentor. Finally, the expressed KsLX3-SIase was purified and was found to have maximum activity at 45 °C and pH 5.5. Its *K*_m_ for sucrose was 267.6 ± 18.6 mmol/L, and its *k*_cat_/*K*_m_ was 10.1 ± 0.2 s^−1^mM^−1^. These findings demonstrated an efficient expression of SIase in *B. subtilis,* and this is thought to be the highest level of SIase produced in a food-grade bacteria to date.

## 1. Introduction

Isomaltulose is a sucrose isomer with similar physical properties and flavor to sucrose [1,2]. Isomaltulose has been recognized as a functional sugar with superior properties, including high acid stability, a low glycemic index, and non-cariogenicity [2]. It is also beneficial for the growth of bifidobacteria in the human intestine [3]. In addition, isomaltulose is non-toxic and non-mutagenic according to Ame’s test [4]. Currently, isomaltulose is increasingly used as a substitute for sucrose in food processing and as a raw material for the production of surfactants [3,4]. Isomaltulose occurs naturally in trace quantities in honey and sugar cane. Its chemical synthesis is complex. Currently, isomaltulose is produced commercially from sucrose using a bacterial strain that naturally expresses sucrose isomerase (SIase) [5]. SIase uses sucrose as a substrate and converts it mainly to isomaltulose, together with a small proportion of trehalulose, and trace byproducts, including glucose and fructose [5,6].

SIase has been discovered in several microbial strains, including *Erwinia rhapontici*, *Serratia plymuthica*, *Klebsiella* sp, *Protaminobacter rubrum*, and *Pantoea dispersa* [5]. The low yield of SIase and a lack of food-safe genetic background have impeded the industrial production of SIase using natural strains. Therefore, the industrial production of isomaltulose would benefit from a strain capable of expressing SIase efficiently using a food-grade heterologous expression system. *Escherichia coli* (*E.coli*) is the most commonly used host for heterologous protein expression, because of its rapid growth rate, high-level of expression of the recombinant protein, and ease of genetic manipulation. SIases from various microbes (including *Protaminobacter rubrum* CBS 547.77, *Pantoea dispersa* UQ68J, and *Klebsiella* sp. LX3) have been expressed in *E.coli* as an intracellular enzyme and the expression of SIase in *E.coli* has been enhanced using response surface methodology [7,8,9,10]. However, the soluble form of SIase in *E.coli* is typically found in very low quantities, and numerous forms of misfolded enzyme have been detected in inclusion bodies [8,11]. In addition, the expressed SIase in *E.coli* is localized in the cytoplasm, necessitating a costly and time-consuming purification process [9,12]. Furthermore, *E.coli* is unsuitable for the production of SIase in the food industry because of safety concerns raised by the synthesis of endotoxins. Therefore, heterologous expression of SIase, achieved using food-grade strains, would be more desirable. SIase is currently expressed in food-grade strains such as *Lactococcus lactis*, *Yarrowia lipolytica*, and *Bacillus subtilis* [5]. SIase from *Enterobacter* sp. FMB-1 has been expressed and secreted in *Lactococcus lactis* MG1363, with an activity of less than 3.0 U/mL [13]. SIase from *Erwinia rhapontici* NX-5 has been expressed as an intracellular form in *Bacillus subtilis* WB800 using the plasmid pHA01, and a 5.2 U/mL whole-cell activity was achieved after 35 h of fermentation performed in a 7.5-L fermentor [14]. SIase from *Pantoea dispersa* UQ68J has been expressed in *Yarrowia lipolytica,* and an activity level of 6.2 U/mL was detected in the culture supernatant [15]. To date, the heterologous expression of SIase in food-grade strains has still yielded a low level of the enzyme [5].

*Bacillus subtilis*, a well-known generally recognized as safe (GRAS) bacterium, which is a designation by the United States Food and Drug Administration (US-FDA) that a substance added to food is considered safe by experts under the conditions of its intended use. *Bacillus subtilis* has been utilized extensively in commercial enzyme production. *Bacillus subtilis* possesses a number of advantages, including nonpathogenicity, suitability for large-scale fermentation, and a diverse toolkit for genetic manipulation [16,17,18,19,20]. In addition, various signal peptides and promoters can be used to achieve effective heterologous protein expression in *Bacillus subtilis* [16,21,22]. Signal peptides have been shown to have a great influence on the secretion of enzymes in *Bacillus subtilis*. The optimization of the signal peptide of a specific enzyme was generally carried out using high-throughput screening of the signal peptide library or semi-rational design based on the signal peptide composition [23,24]. Using an optimized CitH signal peptide identified in a signal peptide library, the activity of a thermostable glucosidase was increased from 1.00 to 5.20 U/mL in the culture supernatant, by expressing the enzyme as a secretory form in *B. subtilis* [25]. An increase in the titer of an alkaline polygalacturonate lyase produced in *B. subtilis* was achieved using a semi-rational strategy with six signal peptides [24]. However, a single signal peptide cannot be optimal for all heterologous proteins. Therefore, it is necessary to screen for a suitable signal peptide for a specific target protein.

To overcome the bottlenecks resulting from inadequate SIase expression and secretion in food-grade strains, we optimized the regulatory element of the signal peptide in the expression plasmid, to improve the expression of SIase from *Klebsiella* sp.LX3 (referred to as KsLX3-SIase) in *Bacillus subtilis*. Using a semi-rational strategy, the candidate signal peptides were selected and screened, yielding an optimized expression cassette. Using a fed-batch fermentation, the expression of KsLX3-SIase was further enhanced. The recombinant KsLX3-SIase was subsequently purified and characterized. The findings of this study could provide a highly efficient and safe system for the expression of SIase, facilitating large-scale production and utilization of SIase.

## 2. Materials and Methods

### 2.1. Strains, Vectors, and Reagents

The gene encoding SIase from *Klebsiella* sp.LX3 (GenBank accession number AY040843) was cloned and stored in our laboratory. The protease-defective derivative of strain 168, *Bacillus subtilis* WB800N, and the expression vector pHT254 were obtained from AtaGenix Laboratories Co., Ltd. (Wuhan, China). PrimeSTAR HS DNA polymerase, DNA, and protein molecular weight markers were obtained from Takara (Dalian, China). A Plasmid Extraction Kit and DNA Gel Purification Kit were purchased from Sangon Biotech (Shanghai, China). Ni-NTA affinity chromatography resin was purchased from Sangon Biotech (Shanghai, China). HPLC grade acetonitrile was purchased from Merck (Darmstadt, Germany). Hypersil APS-2 NH_2_ column (4.6 × 250 mm, 5 μm) was purchased from ThermoFisher Scientific (Bellefonte, PA, USA). All other chemical reagents used in this study were of analytical grade, unless otherwise stated. MilliQ water was used to prepare buffers and all of the buffers were filtered through a 0.22-μm syringe filter, before use. Luria–Bertani (LB) medium (tryptone 10.0 g/L, yeast extract 5.0 g/L, and NaCl 10.0 g/L) were used to culture *E.coli* and *Bacillus subtilis* strains. For protein expression, *B. subtilis* was cultured in 2 × Yeast Extract and Tryptone (YT) medium (tryptone 16.0 g/L, yeast extract 10.0 g/L, and NaCl 5.0 g/L). The fermentation medium consisted of 8 g/L glycerol, 8.5 g/L soybean peptone, 8.5 g/L beef paste, and 5 μg/mL chloramphenicol. The feeding solution contained 150 g/L glucose, 100 g/L soybean peptone, and 50 g/L beef paste.

### 2.2. Plasmid Construction

Different signal peptides were fused to the N-terminus of SIase. These signal peptides included WapA, Bpr, LipA, Yjfa, Mpr, SacB, YvgO, AmyQ, YfhK, AmyE, WprA, Vpr, and AprE. The gene encoding each signal peptide-SIase fusion protein was synthesized and inserted into the pHT254 vector at the *Bam*HI/*Aat*II sites, to generate different pHT254-*Pgrac100-signal peptide-SIase* plasmids, which were constructed by Synbio Technologies (Suzhou, China). All DNA manipulations and molecular cloning experiments were performed following the conventional protocols [26]. *B. subtilis* WB800N was transformed with these plasmids to obtain a recombinant strain capable of producing SIase fused to a signal peptide. *B. subtilis* competent cells were prepared and transformed with the plasmids, as previously described [27]. The transformants harboring different pHT254 vectors were selected on LB-agar plates supplemented with 5 μg/mL chloramphenicol as a selection marker. Positive colonies were confirmed using colony PCR and DNA sequencing (Beijing Genomics Institution, Beijing, China).

### 2.3. Expression of KsLX3-SIase in Bacillus subtilis

To screen the signal peptides for efficient expression of KsLX3-SIase, single colonies harboring various pHT254-*Pgrac100-signal peptides-SIase* were inoculated into LB medium supplemented with 5 μg/mL chloramphenicol and incubated for 12 h at 37 °C. A 0.4-mL sample of the seed culture was then transferred into a 100-mL Erlenmeyer flask containing 20 mL of 2 × YT medium supplemented with 5 μg/mL chloramphenicol and incubated at 37 °C. For inducible expression under the control of the Pgrac100 promoter, IPTG was added to the culture to a final concentration of 1 mM when the OD_600_ reached 0.8–1.0. After the addition of IPTG, the culture was further incubated at 30 °C for 24 h, to induce the expression of SIase.

### 2.4. Fed-Batch Fermentation

Fed-batch fermentation was conducted at 37 °C in a 5-L bioreactor. The seed cultures were prepared by inoculating a single colony of the engineered *B. subtilis* strain into 5 mL of 2 × YT medium supplemented with 5 μg/mL chloramphenicol, followed by 8 h of incubation at 37 °C. A 2-mL sample of the resulting seed culture was transferred to 200 mL of 2 × YT medium containing 5 μg/mL chloramphenicol and incubated at 37 °C for 8 h. The resulting culture was added to 1.8 L of fermentation medium in the fermenter, to bring the total volume of the cultures to 2 L. Ammonia solution and phosphoric acid were used to maintain the pH of the culture at 7.0. The dissolved oxygen (DO) concentration was maintained at 30%. A feeding solution was added to the fermentation culture when the dissolved oxygen concentration rose rapidly. SIase expression was initiated by the addition of IPTG to a final concentration of 1 mM when the OD_600_ of the culture reached 10–12, and the fermentation temperature was adjusted to 30 °C for the entire duration of SIase expression. Samples of the culture were taken at specific time points and centrifuged at 10,000× *g* for 15 min, and the supernatant was analyzed for SIase activity, to assess the expression level of SIase.

### 2.5. Purification of KsLX3-SIase

The culture supernatant was filtered through a 0.45-μm filter, concentrated, and then subjected to immobilized metal ion affinity chromatography (IMAC) using a nickel-nitrilotriacetic acid (Ni-NTA) column coupled to an AKTA purifier 100 system (GE Healthcare, Umeå, Sweden). The column was loaded with the sample and then washed with wash buffer (50 mM PBS, 50 mM imidazole, pH 8.0), to remove nonspecific bound proteins. The flow rate was maintained at a constant 1 mL/min. The column was then eluted with elution buffer (50 mM PBS, 100 mM imidazole, pH 8.0) to remove the bound SIase, which was then dialyzed against 20 mM pH 8.0 Tris-HCl buffer using a 10 kDa cut-off filter and then concentrated using ultrafiltration. The purified SIase was subjected to SDS-PAGE using 12% gel and its protein concentration was determined using the BCA method, with bovine serum albumin (BSA) as a standard.

### 2.6. Characterization of KsLX3-SIase

#### 2.6.1. Assay of the SIase Enzyme Activity

SIase activity was assayed by measuring the conversion of sucrose to isomaltulose. In this assay, 400 μL of 50 mM citric acid-sodium phosphate buffer (pH 6.0) solution containing 40 g/L sucrose was mixed with 100 μL of diluted SIase and incubated at 45 °C for 15 min. The reaction was terminated by boiling the sample for 15 min. After that, the sample was centrifuged at 10,000× *g* for 15 min and the resulting supernatant was filtered through a 0.22-μm membrane filter. The amount of isomaltulose in the filtrate was then determined by HPLC analysis using a Hypersil APS-2 column coupled to an Agilent 1260 system equipped with a refractive index detector (Agilent, Waldbronn, Germany). The column was eluted with a mobile phase, consisting of acetonitrile and water (80:20 *v*/*v*), at a flow rate of 1.0 mL/min. One unit of SIase activity was defined as the amount of enzyme that produced 1 μmol isomaltulose per min under the specified conditions.

#### 2.6.2. Effects of Temperature and pH on SIase Activity

The optimum temperature of SIase was determined by measuring its activity at a temperature ranging from 20 to 60 °C. To determine the thermostability of SIase, the enzyme was pre-incubated at various temperatures, ranging from 35 °C to 60 °C, for 30 min, and the residual SIase activity was then measured under the optimal conditions established. To determine the optimum pH for SIase activity, the assay was performed in 50 mM citric acid-sodium phosphate buffer, with pH ranging from 4.0 to 8.0, at 45 °C for 15 min. To test the stability of SIase at different pH values, the purified enzyme was pre-incubated at 25 °C in a pH ranging from 4.0 to 8.0 for 24 h, and the residual activity was then measured under optimal conditions.

#### 2.6.3. Determination of Kinetic Parameters

To determine the kinetic parameters, the purified SIase was incubated at 45 °C for 15 min in a 50 mM citric acid-sodium phosphate buffer (pH 5.5) containing sucrose ranging from 8 to 640 mM. The *K*_m_, *V*_max_, and *k*_cat_ were determined from Michaelis-Menten plots using (GraphPad Prism 9, GraphPad Software, San Diego, USA).

#### 2.6.4. Storage Stability of the SIase

To determine the storage stability of SIase, the enzyme was kept at 4 °C for 14 days. The SIase activity of samples taken at specific time intervals was determined under optimal conditions. Residual activity was calculated with reference to the initial activity.

#### 2.6.5. Time Course of Production of Isomaltulose

The time course reaction was conducted in a biotransformation mimic system (10 mL capacity), using a 50 mM citric acid-sodium phosphate buffer (pH 5.5) solution containing 80 g/L, 120 g/L, and 160 g/L sucrose. SIase concentration was set at 8 μg/mL. The reaction was conducted at 25 °C for 6 h, with shaking at 200 rpm. At specified intervals, aliquots of the reaction mixture were taken, and the isomaltulose produced was quantified by HPLC, with the same parameters as described in detail in Section 2.6.1.

## 3. Results and Discussion

### 3.1. Optimization of Signal Peptides for SIase Expression

**Figure 1:**
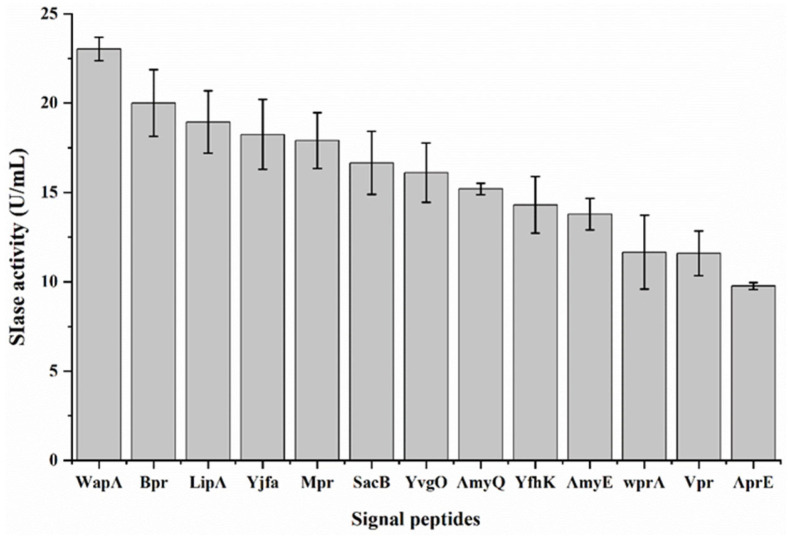
Effects of different signal peptides on the secretion of KsLX3-SIase expressed in *B. subtilis* WB800N. Extracellular SIase activity was assayed with sucrose as the substrate. The production of isomaltulose from sucrose was determined by HPLC. Data are the means ± SD from triplicate samples.

Insufficient SIase secretion by food-grade strains has impeded the industrial production of isomaltulose. The signal peptide is crucial for the expression and secretion of the target protein. Recent research has demonstrated that an optimal signal peptide for a protein can be determined by a semi-rational design, based on signal peptide composition [24]. Signal peptides with a more positively charged N-domain, a more hydrophobic H-domain, and a more conserved C-domain can better facilitate the secretion of the target protein [24,28,29]. Based on the literature [28], thirteen signal peptides were selected as candidates for this study (Table 1). These signal peptides were placed upstream of the KsLX3-SIase coding sequence, which was under the control of a strong IPTG-inducible promoter Pgrac100 [30], and the resulting recombinant plasmids were separately introduced into *Bacillus subtilis* WB800N for the expression of KsLX3-SIase. The signal peptide WapA was found to yield the highest expression of KsLX3-SIase, as detected by measuring its activity (23.0 U/mL) in the culture supernatant (Figure 1). In addition, each of the selected signal peptides was able to direct the secretion of KsLX3-SIase to the outside of the cell. WapA is a native twin-arginine signal peptide of *B. subtilis*, suggesting that KsLX3-SIase could be secreted through both the Tat and Sec pathways. The result indicated that screening for an optimal signal peptide is necessary, to ensure the efficient secretion and high yield of a specific protein expressed in *B. subtilis*. Therefore, all subsequent experiments aiming to optimize the expression of KsLX3-SIase were performed with the WapA signal peptide.

### 3.2. Production of SIase Using a Fed-Batch Strategy

**Figure 2:**
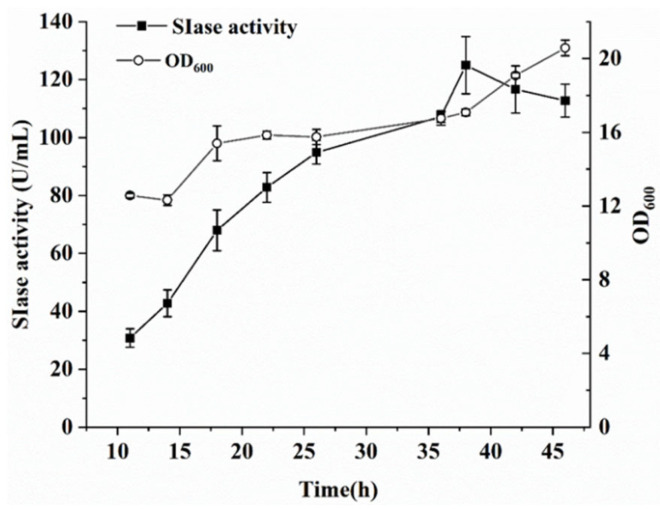
Fed-batch fermentation of the recombinant WB800N/pHT254-*Pgrac100-WapA-SIase* in a 5-L fermenter. Extracellular SIase activity (filled square), biomass (open cycle). Data are the means ± SD from triplicate samples.

**Figure 3:**
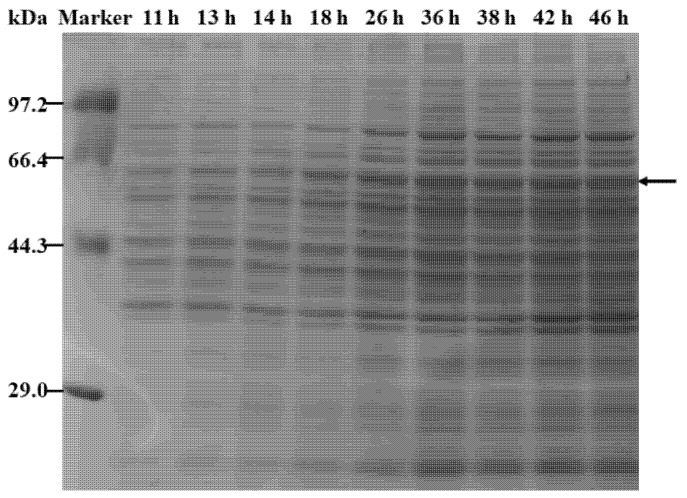
SDS-PAGE analysis of SIase in the culture supernatant of the fed-batch fermentation.

Using a food-grade expression system to produce SIase would facilitate its widespread application in the food industry. Using fed-batch fermentation carried out in a 5 L fermenter, the production capacity of the recombinant strain WB800N/pHT254-*Pgrac100-WapA-SIase* was determined. Carbon and nitrogen resources were supplemented, so that cell growth and SIase expression could be effectively controlled. During the fermentation process, the rotatory speed was coupled to the DO, to maintain a DO level of 30%. The biomass (OD_600_) and SIase activity in the extracellular medium were examined. As shown in Figure 2, the extracellular SIase activity peaked at 125.0 U/mL after 38 h of fermentation. The results of SDS-PAGE confirmed that the amount of secreted SIase continuously increased during the fermentation process (Figure 3).

The results seemed to validate the production of SIase using the food-grade strain. In *Lactococcus lactis* MG1363, SIase consisting of heterogenous forms was secreted with an activity lower than 3 U/mL [13]. A food-grade yeast strain, *Yarrowia lipolytica*, has also been used to express SIase, achieving a SIase activity of 49.3 U/mL in the culture supernatant [12]. However, the cultivation of yeast is rather lengthy (more than 72 h) and costly. Compared with these strains, *B. subtilis* offers several significant advantages, including a high efficiency of secretion for the target protein within a shorter fermentation time. In a previous study, when SIase from *Erwinia rhapontici* NX-5 was expressed in *B. subtilis* WB800 under the control of the Pgrac promoter, the enzyme was mainly found in intracellular form, with total SIase activity in the cell extract reaching 5.2 U/mL of culture in a 7.5-L fermenter [14]. In our study, using the optimal signal peptide WapA, the activity of KsLX3-SIase in the culture medium was determined to be 125.0 U/mL, which is the highest level of SIase activity produced in a food-grade bacteria to date. The increased level of KsLX3-SIase in our study demonstrated the necessity of optimizing a signal peptide for efficient expression of a specific protein. In addition, the extracellular secretion of SIase in this study could simplify the purification process, which would be desirable for industrial production of SIase. The fermentation process could be enhanced by systematically optimizing the feeding strategies [16].

### 3.3. Enzymatic Properties of the Purified KsLX3-SIase

**Figure 4:**
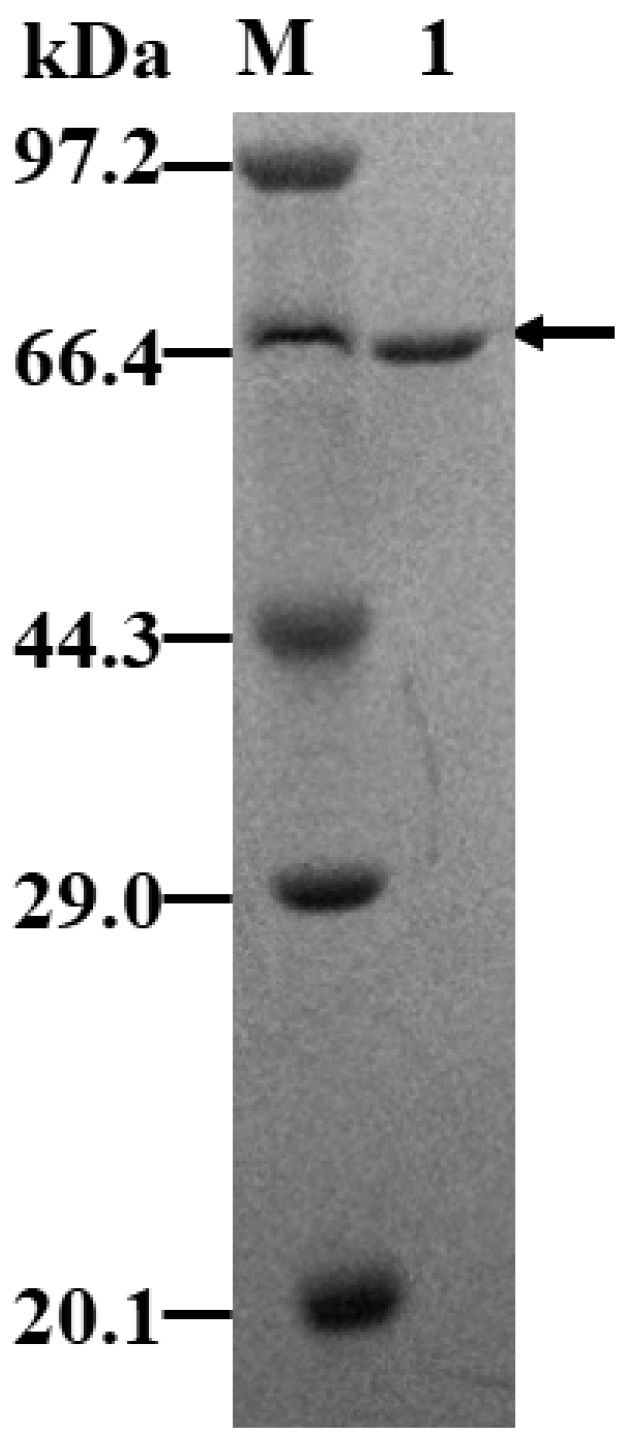
SDS-PAGE analysis of KsLX3-SIase expressed in *B. subtilis* and purified using Ni-NTA chromatography. M: protein marker, 1: purified SIase.

To investigate the enzymatic properties of KsLX3-SIase expressed in *B. subtilis*, the recombinant KsLX3-SIase was purified and characterized. The expressed KsLX3-SIase was purified by affinity chromatography using Ni-NTA. SDS-PAGE analysis of the purified KsLX3-SIase revealed a single band with a molecular mass of approximately 66 kDa (Figure 4). Previously, the expression of the recombinant SIase has mainly been carried out in *E. coli*, and only a low level of SIase was expressed, as an intracellular protein. Using *B. subtilis* as a host could, therefore, greatly facilitate the secretion of the SIase into the medium of the culture, simplifying its purification.

**Figure 5:**
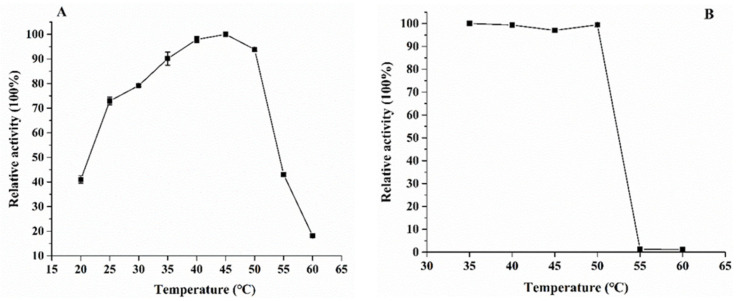

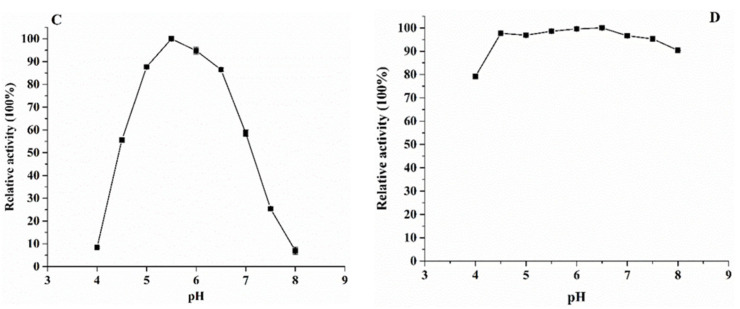
Effects of temperature and pH on the properties of KsLX3-SIase. Effect of temperature on enzyme activity (**A**) and stability (**B**). Effect of pH on enzyme activity (**C**) and stability (**D**). Data are the means ± SD from triplicate samples.

The effect of temperature on the activity of the purified KsLX3-SIase was measured at temperatures ranging from 20 to 60 °C. KsLX3-SIase exhibited a maximum activity at 45 °C, with more than 90% of its maximal activity occurring at 35 °C to 50 °C, but its activity decreased sharply above 50 °C (Figure 5A). The optimal temperature of KsLX3-SIase was therefore slightly higher than that of most other SIases, such as those of *Erwinia rhapontici* NX-5, *Klebsiella pneumoniae* NK33, and *Pantoea dispersa* UQ68J, which have an optimal activity at 30–35 °C [8,31,32]. The thermostability of KsLX3-SIase was evaluated at temperatures ranging from 35 °C to 60 °C. KsLX3-SIase was found to retain more than 97% of its maximum activity after incubation for 30 min at temperatures between 35 °C and 50 °C, indicating that the enzyme exhibited good thermostability at these temperatures (Figure 5B). The KsLX3-SIase began to denature at temperatures above 50 °C.

The effect of pH on the activity of KsLX3-SIase was determined at a pH ranging from 4.0 to 8.0. The enzyme was found to have optimal activity at pH 5.5, but it exhibited good activity in the pH range of 5.0 to 6.5 (Figure 5C). The activity decreased rapidly at a pH greater than 6.5 or less than 5.0. Most recombinant SIases tend to have an optimal pH between 5.0 and 6.0 [5], which is similar to what was determined for KsLX3-SIase. The results of the pH stability analysis indicated that KsLX3-SIase was stable over a wide range of pHs at 25 °C (Figure 5D). After incubation at pHs between 4.5 and 8.0 for 24 h, the enzyme retained more than 90% of its maximum activity.

**Figure 6:**
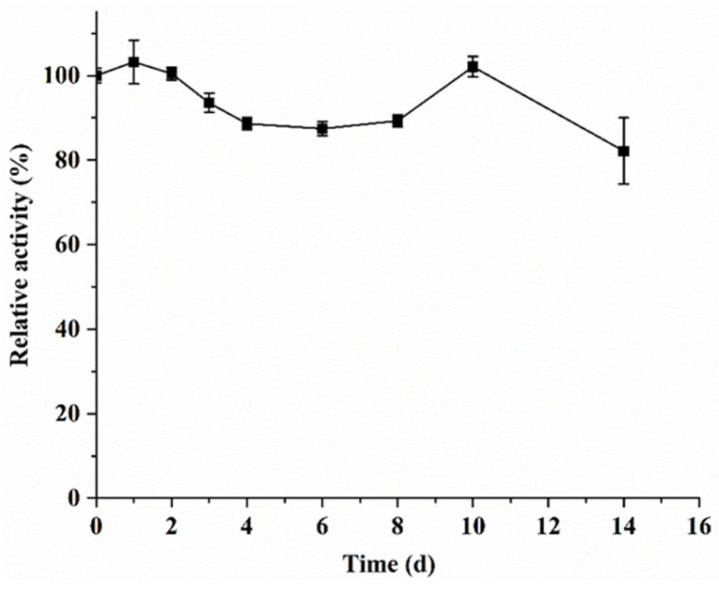
Storage stability of KsLX3-SIase. Data are the means ± SD from triplicate samples.

The specific activity of purified KsLX3-SIase under optimal reaction conditions was 594 U/mg. Using a Michaelis–Menten plot (Figure S1), the *K*_m_ and *k*_cat_/*K*_m_ of KsLX3-SIase were determined to be 267.6 ± 18.6 mM and 10.1 ± 0.2 s^−1^mM^−1^, respectively. The *K*_m_ value was comparable to that of SIase from *Erwinia rhapontici* NX-5 [31]. In addition, the *k*_cat_/*K*_m_ of KsLX3-SIase was higher than that of *K.planticola* UQ14 S, *Pantoea dispersa* UQ68J, and *Erwinia* sp. Ejp617 [5,33], indicating its high catalytic efficiency, which is advantageous for practical applications. The storage stability of KsLX3-SIase was examined by measuring the residual enzyme activity upon storage at 4 °C for 14 days. KsLX3-SIase maintained 82% of its initial activity after 14 days, demonstrating its superior stability over a long storage period (Figure 6).

**Figure 7:**
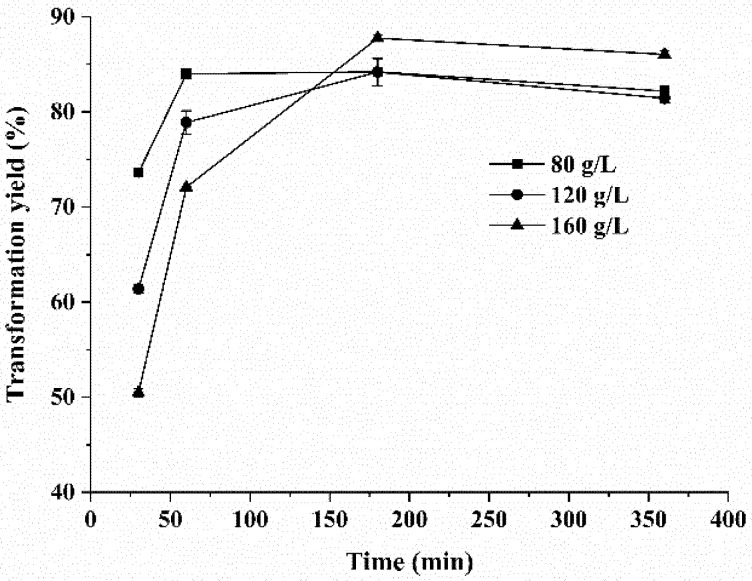
Time course of isomaltulose production catalyzed by KsLX3-SIase. Data are means ± SD from triplicate samples.

The time-course reaction catalyzed by KsLX3-SIase produced in *B. subtilis* was investigated by employing various sucrose concentrations. As depicted in Figure 7, the maximum yields of isomaltulose were 84.2 ± 0.3%, 84.2 ± 1.4%, and 87.8 ± 0.3% when the sucrose concentrations used were 80 g/L, 120 g/L, and 160 g/L, respectively, demonstrating that a high yield of isomaltulose could be obtained with this enzyme in the presence of a high sucrose concentration. In addition, the rate of isomaltulose yield was rapid during the initial 60 min but then decreased with further culturing time, for all three concentrations of sucrose. This phenomenon might be explained by the inhibitory effect of the by-products, glucose and fructose, or the reduced stability of SIase in the high viscosity of the reaction mixture. A high yield of isomaltulose is necessary for its cost-effective industrial production. The yield we obtained for KsLX3-SIase was greater than that reported for the SIase from *Enterobacter* sp. The yields of isomaltulose achieved with FMB-1 expressed in *Lactococcus lactis* MG1363 and *Saccharomyces cerevisiae* EBY100 were 72% and 7.4%, respectively [13,34]. In addition, the yield of isomaltulose produced by KsLX3-SIase expressed in *B. subtilis* was comparable to the yields of isomaltulose obtained with other SIases expressed in several different organisms (Table 2). For example, the SIase from *Erwinia rhapontici* NX-5, which was expressed in *Bacillus subtilis* spores gave a yield of 92%, whereas the SIase from *Pantoea dispersa* UQ68J expressed in *Yarrowia lipolytica* gave a yield of 93% [35,36].

## 4. Conclusions

In this study, high-level extracellular production of a SIase from *Klebsiella* sp.LX3 in *Bacillus subtilis* was achieved by optimizing the signal peptides. The signal peptide WapA had the highest secretion efficiency for KsLX3-SIase and resulted in an activity level of 23.0 U/mL in the culture medium. Using a fed-batch strategy, the extracellular KsLX3-SIase activity in the culture was increased to 125.0 U/mL, which is the highest level of SIase activity expressed in a food-grade bacteria to date. In addition, KsLX3-SIase exhibited an optimal activity at 45 °C and pH 5.5, as well as a good catalytic efficiency and a high yield of isomaltulose under various sucrose concentrations. These results demonstrated an efficient expression of SIase in *B. subtilis*, laying the foundation for scaled-up production and industrial application of SIase.

## Figures and Tables

**Table 1:** Comparison of various signal peptide sequences used for KsLX3-SIase expression.

Signal Peptide	Sequence	Secretory Pathway	Charged N-Region	Hydrophobic Amino Acid
AmyE	MFAKRFKTSLLPLFAGFLLLFHLVLAGPAAASA	Sec	3	20
AprE	MRSKKLWISLLFALTLIFTMAFSNMSVQA	Sec	3	15
AmyQ	MIQKRKRTVSFRLVLMCTLLFVSLPITKTSA	Sec	4	12
Bpr	MRKKTKNRLISSVLSTVVISSLLFPGAAGA	Sec	5	13
Mpr	MKLVPRFRKQWFAYLTVLCLALAAAVSFGVPAKA	Sec	4	17
Vpr	MKKGIIRFLLVSFVLFFALSTGITGVQAAPA	Sec	3	16
YjfA	MKRLFMKASLVLFAVVFVFAVKGAPAKA	Sec	3	16
YvgO	MKRIRIPMTLALGAALTIAPLSFASA	Sec	3	15
SacB	MNIKKFAKQATVLTFTTALLAGGATQAFA	Sec	3	11
YfhK	MKKKQVMLALTAAAGLGLTALHSAPAAKA	Sec	3	17
LipA	MKFVKRRIIALVTILMLSVTSLFALQPSAKAA	Tat	2	15
WapA	MKKRKRRNFKRFIAAFLVLALMISLVPADVLA	Tat	6	16
WprA	MKRRKFSSVVAAVLIFALIFSLFSPGTKAAA	Tat	4	16

**Table 2:** Conversion of sucrose to isomaltulose using SIases from various microbes with different expression systems.

SIase Source	Expression System	Conversion Conditions	Yield of Isomaltulose (%)	Reference
Enterobacter sp. FMB-1	Lactococcus lactis MG1363	pH 6.0, 30 °C	72	[13]
Enterobacter sp. FMB-1	Saccharomyces cerevisiae EBY100	pH 6.0, 45 °C	7.4	[34]
Erwinia rhapontici NX-5	Bacillus subtilis 168	pH 6.0, 30 °C	92	[35]
Pantoea dispersa UQ68J	Yarrowia lipolytica	pH 6.0, 30 °C	93	[36]
Klebsiella sp.LX3	Bacillus subtilis WB800N	pH 5.5, 25 °C	87.8	This study

## Data Availability

The data that support the findings of the present study are available from the corresponding author on reasonable request.

## Supplementary Materials

The following supporting information can be downloaded at: https://www.mdpi.com/article/10.3390/foods11162468/s1, Figure S1: Michaelis–Menten plot for purified KsLX3-SIase with sucrose as a substrate.
