# Supplementary material for: Enhanced Extracellular Production and Characterization of Sucrose Isomerase in Bacillus subtilis with Optimized Signal Peptides
Source: Foods. 2022 Aug 16;11(16):2468. doi: 10.3390/foods11162468 (PMC9407248; doi:10.3390/foods11162468)
Supplement: Supplementary file 1 [file foods-11-02468-s001.zip › foods-1851628-supplementary.pdf]

foods

MDPI

Article

Enhanced Extracellular Production and Characterization of Sucrose Isomerase in *Bacillus subtilis* with Optimized Signal Peptides

Dan Guo [1,†], Mingyu Li [1,†], Mengtong Jiang [1], Guilong Cong [1], Yuxin Liu [1], Conggang Wang [1,*], Xianzhen Li [1]

[1] School of Biological Engineering, Dalian Polytechnic University, Dalian 116034, People's Republic of China; GD_1093753211@163.com (D.G.); limingyu970525@163.com (M.L.); JMT3896@163.com (M.J.); congguilong66@163.com (G.C.); yxliu0727@163.com (Y.L.)

* Correspondence: wangcg@dlpu.edu.cn; Tel.:+86-411-86318692

† All authors contributed equally

Citation: Lastname, F.; Lastname, F.; Lastname, F. Title. *Foods* **2022**, *11*, 2468. https://doi.org/10.3390/10.3390/foods11162468

Academic Editor: Firstname Lastname

Received: date

Accepted: date

Published: date

Publisher's Note: MDPI stays neutral with regard to jurisdictional claims in published maps and institutional affiliations.

Copyright: © 2022 by the authors. Submitted for possible open access publication under the terms and conditions of the Creative Commons Attribution (CC BY) license (https://creativecommons.org/licenses/by/4.0/).

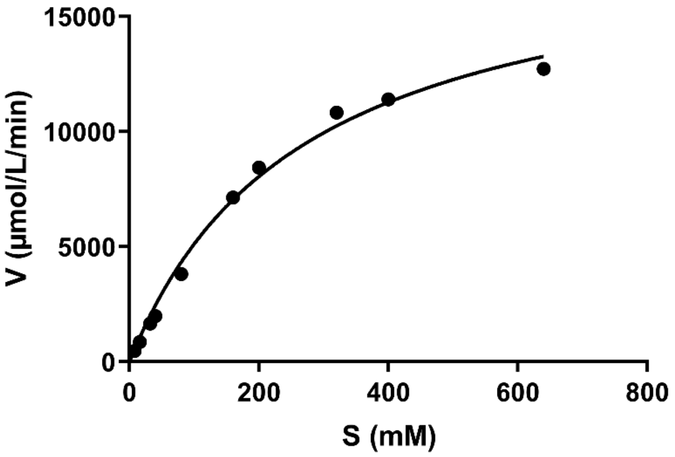

Figure S1. Michaelis-Menten plot for purified KsLX3-SIase with sucrose as a substrate
